# Cerebrospinal fluid positive for SARS-CoV-2 associated with neurological manifestations and proteome alterations in a multiple myeloma patient

**DOI:** 10.1007/s10072-026-09471-w

**Published:** 2026-10-19

**Authors:** Jayr Schmidt-Filho, Thais Rodrigues B. Geraldo, Victor C. Carregari, Marina de Mattos Nascimento, Anita Cassoli Cortez, Ramon Teixeira Costa, Ivan Leonardo Avelino Franca-Silva, Israel Tojal da Silva, Marcos Aurélio Peterlevitz, Alessandro S. Farias, José Eduardo Vitorino Galon, Ana Maria Fraga, Celso Francisco Hernandes Granato, Gislaine Cristina Lopes Machado Porto, Diana Noronha Nunes, Dirce Maria Carraro, Kenneth J. Gollob, Emmanuel Dias-Neto, Daniel Martins-de-Souza, Vilma R. Martins, Marjorie Vieira Batista

**Affiliations:** 1 Department of Hematology and Cell Therapy, A.C.Camargo Cancer Center, São Paulo, SP Brazil; 2 Reference Center for Hematologic Neoplasms at the A.C.Camargo Cancer Center, São Paulo, Brazil; 3 Department of Neurology, A.C.Camargo Cancer Center, São Paulo, Brazil; 4 Laboratory of Neuroproteomics, Department of Biochemistry and Tissue Biology, Institute of Biology, University of Campinas (UNICAMP), Campinas, Brazil; 5 Intensive Care Unit, A.C.Camargo Cancer Center, São Paulo, Brazil; 6 Department of Infectious Diseases and Infection Control, A.C.Camargo Cancer Center, R. Prof. Antônio Prudente, 211 - Liberdade, São Paulo, SP 01509-010 Brazil; 7 Laboratory of Computational Biology, A.C.Camargo Cancer Center, São Paulo, Brazil; 8 Experimental Medicine Research Cluster (EMRC), University of Campinas (UNICAMP), Campinas, SP Brazil; 9 Autoimmune Research Laboratory, Department of Genetics, Microbiology and Immunology, Institute of Biology, University of Campinas (UNICAMP), Campinas, Brazil; 10 Laboratory of Virology, Grupo Fleury, São Paulo, Brazil; 11 Department of Imaging, A.C.Camargo Cancer Center, São Paulo, Brazil; 12 Laboratory of Medical Genomics, A.C.Camargo Cancer Center, São Paulo, Brazil; 13 Translational Immuno-oncology Group, International Research Center, A.C. Camargo Cancer Center, São Paulo, Brazil; 14 D’Or Institute for Research and Education (IDOR), São Paulo, Brazil; 15 INCT in Modelling Human Complex Diseases With 3D Platforms (Model3D), São Paulo, Brazil; 16 Department of Cell Biology and Development, Institute of Biomedical Sciences, University of São Paulo, São Paulo, Brazil; 17 National Institute of Science and Technology in Oncogenomic and Pharmaceutical Innovation, São Paulo, Brazil; 18 Translational Immuno-oncology Laboratory, Hospital Israelita Albert Einstein, São Paulo, Brazil; 19 Center for Research in Immuno-oncology (CRIO), Hospital Israelita Albert Einstein, São Paulo, Brazil; 20 Rutgers Cancer Institute, Newark, USA; 21 Division of Cancer Biology, Department of Radiation Oncology, Rutgers New Jersey Medical School, Newark, USA

**Keywords:** Multiple myeloma, COVID-19, SARS-CoV-2, CNS, Neurological, Proteomic, Mass spectrometry, CSF

## Abstract

**Background:**

Although SARS-CoV-2 infection is primarily associated with respiratory symptoms, neurological complications have been increasingly recognized, even in the absence of pulmonary involvement.

**Case presentation:**

We report a case of a 67-year-old woman with a history of multiple myeloma and recent exposure to a COVID-19-positive individual who developed rapidly progressive neurological symptoms, including dysarthria, dysphagia, and hypoesthesia, without any respiratory manifestations. Her condition deteriorated to focal and dyscognitive status epilepticus, requiring mechanical ventilation. Brain MRI showed imaging features consistent with rhombencephalitis. SARS-CoV-2 infection was confirmed through positive qRT-PCR results from both nasopharyngeal swab and cerebrospinal fluid (CSF). Further analysis of the CSF using quantitative shotgun proteomics via mass spectrometry revealed a neuroinflammatory profile indicative of blood–brain barrier disruption and differential expression of several proteins relevant to brain function, including the major prion protein, superoxide dismutase 1 (SOD-1), and other aggregation-prone proteins.

**Conclusions:**

This case supports the hypothesis that SARS-CoV-2 can invade the central nervous system and cause severe neurological impairment, even in the absence of typical respiratory symptoms. The findings underscore the importance of considering SARS-CoV-2 in the differential diagnosis of rapidly progressive neurological syndromes and highlight the need for further investigation into the mechanisms of neuroinvasion and the potential neurotropism of emerging viral variants.

**Supplementary Information:**

The online version contains supplementary material available at https://doi.org/10.1007/s10072-026-09471-w.

## Introduction

SARS-CoV-2, the virus responsible for the COVID-19 pandemic, causes a wide range of clinical manifestations, from mild disease to severe lower respiratory tract infections, often with associated renal, cardiac, and neurological complications. [1] Several factors influence the clinical presentation of COVID-19, including pre-existing comorbidities, immune status, and SARS-CoV-2 genome variants. Among these factors, the impact of immunocompromised states and neoplastic diseases on COVID-19 outcomes remains unclear.

An observational study on solid cancer patients with SARS-CoV-2 infection found COVID-19 mortality rates ranging from 9 to 33%, with ICU mortality rates exceeding 41%. [2] Additionally, data from other respiratory viruses suggest that patients with hematologic malignancies are at higher risk for pulmonary complications [3].

While much remains to be learned about the neurological manifestations of SARS-CoV-2, recent studies have shown that COVID-19 can cause neuropathological changes in the brain, such as microglial nodules, neurophagocytosis, neuronal injury, and endotheliitis. [4] A postmortem study comparing brains from nine critically ill COVID-19 patients with nine healthy controls identified several neuropathological features, including microglial nodules, satellite cell phenomena, edema, focal hemorrhage, infarction, and infiltrating mononuclear cells. Immunostaining revealed widespread activation of microglia and astrocytes, severe blood–brain barrier (BBB) damage, and perivascular infiltration by monocytes and T lymphocytes. Elevated cytokine levels, including IL-6, IL-8, IL-15, and MIP-1b, correlated with BBB damage. Quantitative proteomic analysis identified upregulated proteins involved in immune response, autophagy, and metabolism, while proteins related to brain development and neuroprotection were downregulated, suggesting disruption of neuroprotective mechanisms [5].

**Fig. 1:**
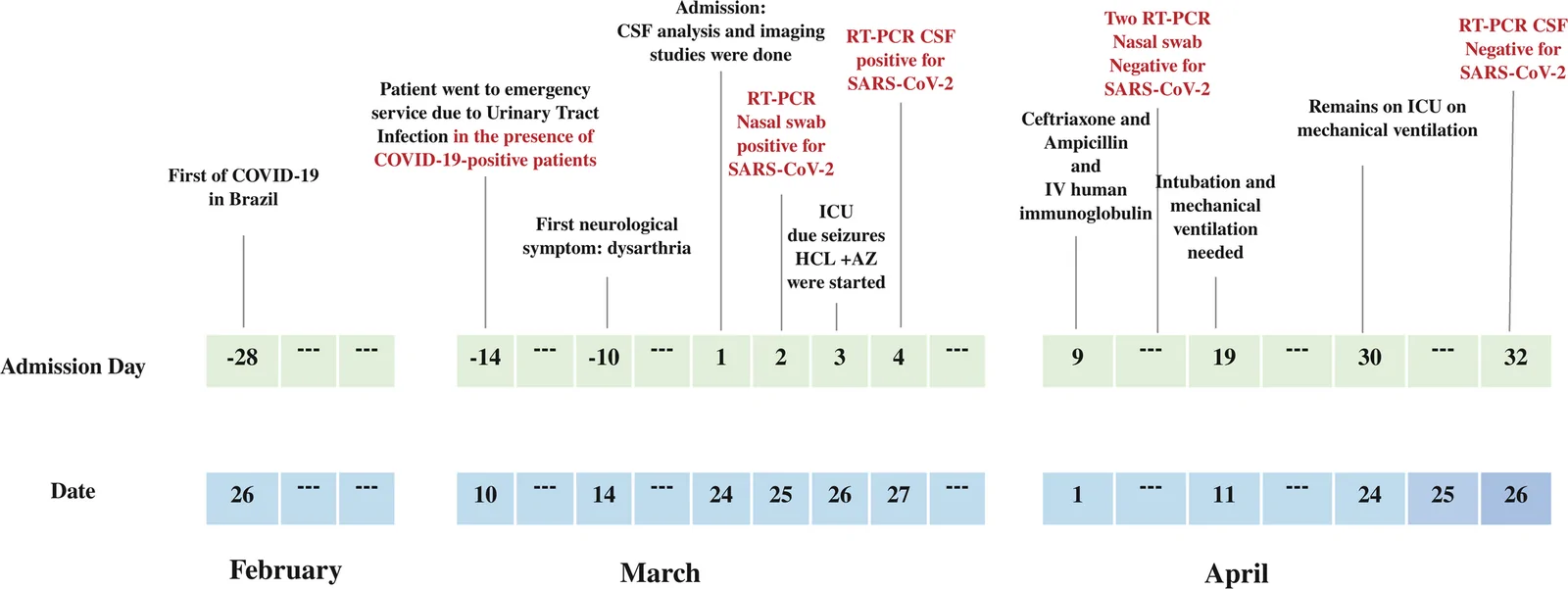
Timeline of key events and sample collection in the study. Timeline of the main events and collection of samples

**Fig. 2:**
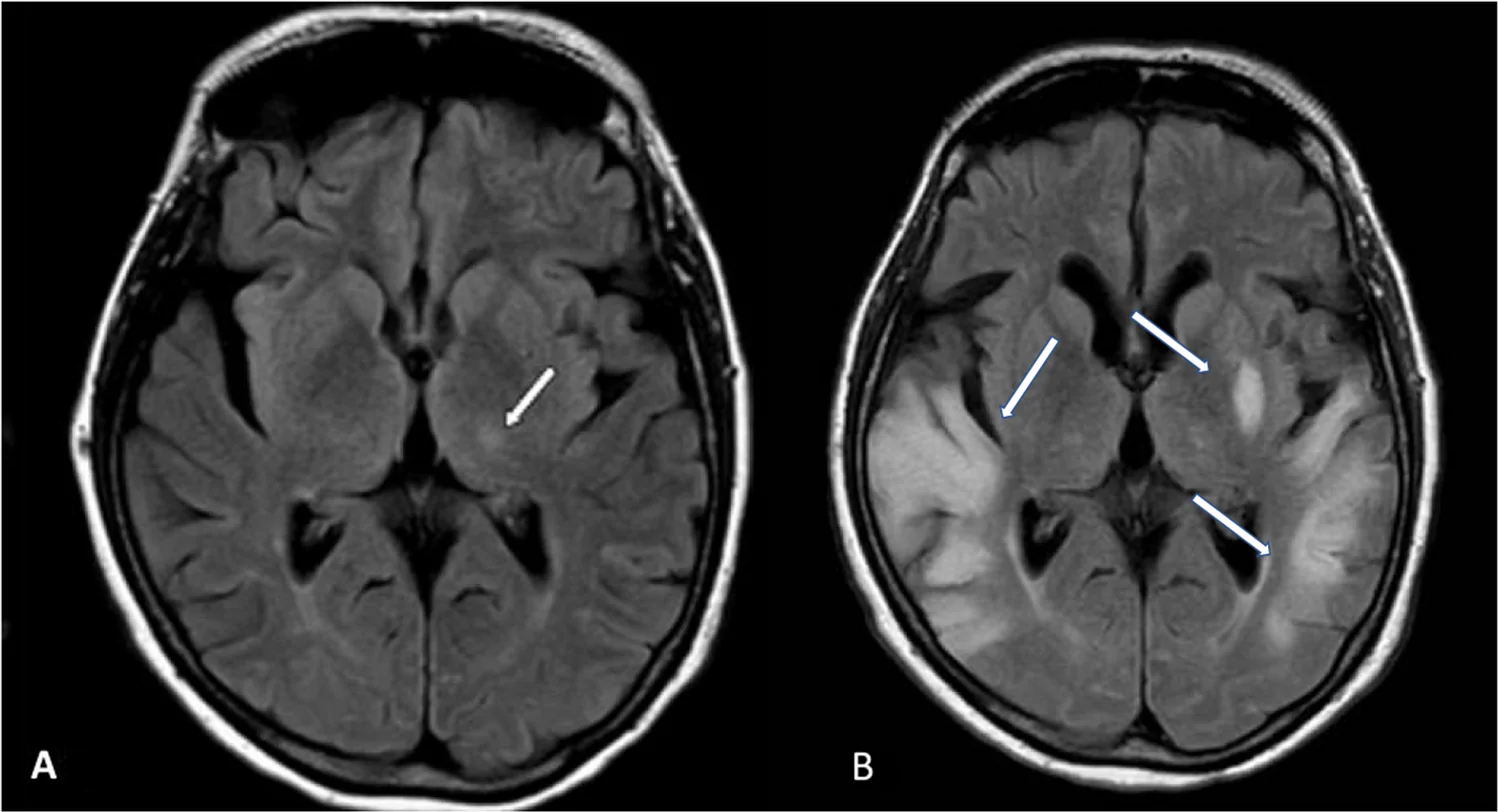
Neuroimaging findings in a patient with multiple myeloma and SARS-CoV-2. Image **A**, T2-weighted image showed hyperintensity signal changes in midbrain and pons (small arrow). Image **B**, Fluid-attenuated inversion recovery image (FLAIR) showed 241 hyperintensity signal changes along the corticospinal tract, more prominent on right (arrows)

Here, we present a case study of a SARS-CoV-2-infected multiple myeloma (MM) patient who developed neurological symptoms, with SARS-CoV-2 detected in the CSF. Mass spectrometry-based quantitative shotgun proteomics was performed to analyze the CSF during and after infection. The results revealed alterations in biological processes and biochemical pathways, indicating that inflammation and neurodegeneration may be linked to the patient's clinical symptoms (Figs. 1 and 2).

## Case report

A 67-year-old woman with a history of MM, diagnosed four years earlier, initially received bortezomib, cyclophosphamide, and dexamethasone followed by autologous stem cell transplantation, achieving a two-year remission. After relapse, she was treated with daratumumab, lenalidomide, and dexamethasone, reaching a very good partial response but developing steroid-induced diabetes and neutropenia.

She presented with a 10-day history of progressive dysarthria evolving to anarthria, dysphagia, right facial hypoesthesia, and hemifacial spasms, without fever or respiratory symptoms. Epidemiological history revealed recent exposure to individuals with suspected COVID-19. On admission neurological examination showed brainstem involvement without pyramidal or cerebellar signs.

Nasopharyngeal swabs tested positive for SARS-CoV-2 and Coronavirus NL63. MRI findings were consistent with rhomboencephalitis, without evidence of MM-related cranial involvement. CSF analysis showed no significant abnormalities, and extensive infectious workup was negative for alternative pathogens. Immunophenotyping excluded central nervous system infiltration by MM.

SARS-CoV-2 was detected in both nasopharyngeal swabs and CSF by qRT-PCR (targeting the nucleocapsid gene with RNAse-P control). Ct values suggested a higher viral load in the CSF (Ct 24.9) compared to nasopharyngeal swabs (Ct 28), consistent with significant central nervous system involvement.

During hospitalization, the patient developed focal seizures progressing to impaired consciousness and required ICU admission. Despite treatment with antiseizure medications, antiviral therapy, antibiotics, intravenous immunoglobulin, and corticosteroids, her condition deteriorated to super-refractory status epilepticus requiring mechanical ventilation.

After 90 days of hospitalization, she was discharged in a minimally conscious state under home care. She died 11 months later due to pneumonia.

## Materials and methods

Written informed consent was obtained from the patient, and the study was approved by the Research Ethics Committee of A.C.Camargo Cancer Center (CEP 2858/20).

### Proteomics

#### Sample preparation

CSF samples were collected at day 4 after admission and presented qRT-PCR positive for SARS-CoV-2 (infection period) and at day 32 with a CSF qRT-PCR negative for SARS-CoV-2 (post-infection period). Fifty μL of the CSF samples were diluted in urea buffer 1:1 v/v (Urea 2 M, Tris–HCl 100 mM and protease inhibitor cocktail) to avoid protease activity, and to clean up the samples and remove salt and other contaminants protein concentration with an Amicon 3KDa filter was performed. Briefly, 100 µl of the CSF solution was added to the filter and the volume was completed until 500µL with urea buffer and centrifuged for 40 min at 4500xg at 8 °C. Proteins were retained in the filter and the remained solution was collected from the filter and used for protein quantification and digestion. Protein digestion from each CSF sample (20 µg) was performed using the FASP protocol. [6] Briefly described in washing steps to buffer exchange in a microcolumn tip with 10 kDa MW cut off, and reduction, alkylation and tryptic digestion performed in column.

#### Liquid chromatography-mass spectrometry

Digested proteins (1 µg) were injected in a nano Acquity UPLC M-Class (Waters Corporation, Milford, MA, USA) liquid chromatograph, which is coupled online to a Synapt G2-Si mass spectrometer (Waters Corporation, Milford, MA, USA). Each peptide sample was separated using a continuous acetonitrile gradient from 3 to 40% (v/v) on C18 columns. Fractionated peptides were ionized by electrospray ionization in positive mode and [Glu 1]-Fibrinopeptide B was used as the lock-mass. MS/MS analyses were performed in data-independent acquisition (DIA) mode using HDMS^E^. Three technical replicates from each sample were performed.

#### Protein identification and quantification

MS/MS spectra were aligned and analyzed with Progenesis QI for Proteomics (version 3.1). Proteins were identified using the reviewed UNIPROT Homo sapiens database (protein ID UP000464024/2021) and using all peptides for protein quantification. The following parameters were used in the identification of peptides/proteins: digestion by trypsin with a maximum of one missed cleavage; maximum protein mass of 400 kDa; false discovery rate (FDR) less than 1%; at least two fragments per peptide, one peptide per protein, and three or more fragments per protein; at least one unique peptide per protein for quantitation; and a mass error ≤ 20 ppm. The expression analysis considered three technical replicates from CSF during infections and CSF post-infection, following the hypothesis that each group is an independent variable. Proteins were considered differentially expressed when the one-way analysis of variance (ANOVA) returned to a *p*-value < 0.05.

#### Systems biology in silico

Differentially expressed proteins exhibiting a fold change of ± 20% were subjected to in silico systems biology analyses using Metascape, incorporating annotations from the KEGG, Reactome, and WikiPathways databases to identify the top 10 enriched pathways, cellular compartments, and disease associations related to the dataset. Cytoscape was employed for protein–protein interaction (PPI) network analysis and figure visualization. Omicscope was used for clustering and statistical analysis [7].

## Results

**Fig. 3:**
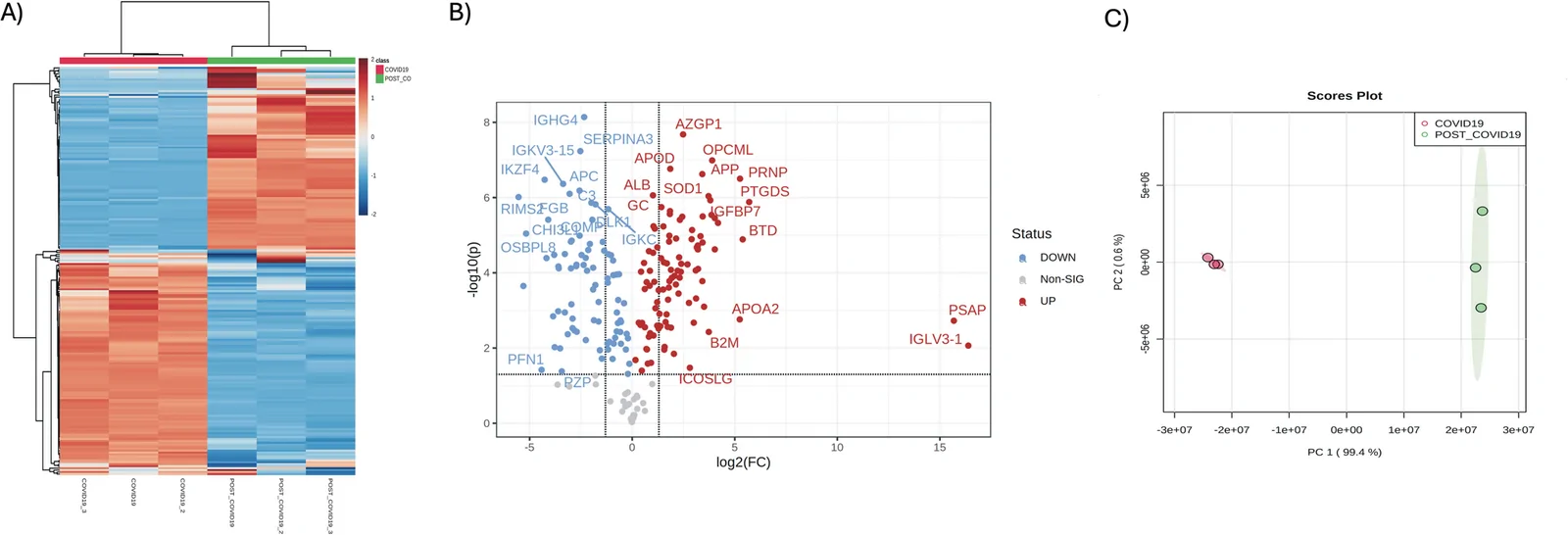
Proteomic analysis of cerebrospinal fluid during the infection (COVID-19) and post-infection (Post-COVID-19) periods. **A**. Heatmap of the 50 most dysregulated proteins, with clustering based on peak intensity during infection (COVID-19) and post-infection (Post-COVID-19) periods. **B**: Volcano plot showing the distribution of upregulated and downregulated proteins between infection and post-infection periods (*p* < 0.05). C: Principal component analysis (PCA) of identified proteins during infection (COVID-19) and post-infection (Post-COVID-19) periods. CSF proteomics from a patient with Multiple Myeloma and SARS-CoV2 at CSF during infection (positive RT-PCR at CSF) and at post-infection (negative RT-PCR at CSF) periods. **A** Heatmap of the 50 most dysregulated proteins based on the peak intensity of each protein. Person’s correlation analysis for clustering of proteins from CSF during infection (COVID19, three technical replicates) and post-infection (POST_COVID19, three technical replicates) periods. **B** Volcano plot showing the distribution of up and down regulated proteins when comparing infection and post-infection periods (*p* < 0.05). C) PCA analysis of all the identified proteins during infection (COVID19) and post-infection (POST_COVID19) periods

CSF proteome analysis identified 211 proteins, of which 184 were differentially expressed, with 99 upregulated and 85 downregulated during infection compared to the post-infection period. This dysregulation in protein expression exhibited a clear proteome signature, which allowed for a distinct separation between the two conditions, as shown in the heatmap (Fig. 3A). The observed changes in protein expression clearly demonstrated a polarity of upregulation and downregulation, further confirmed by Principal Component Analysis (PCA), which distinguished the two groups (Figs. 3B and C).

**Fig. 4:**
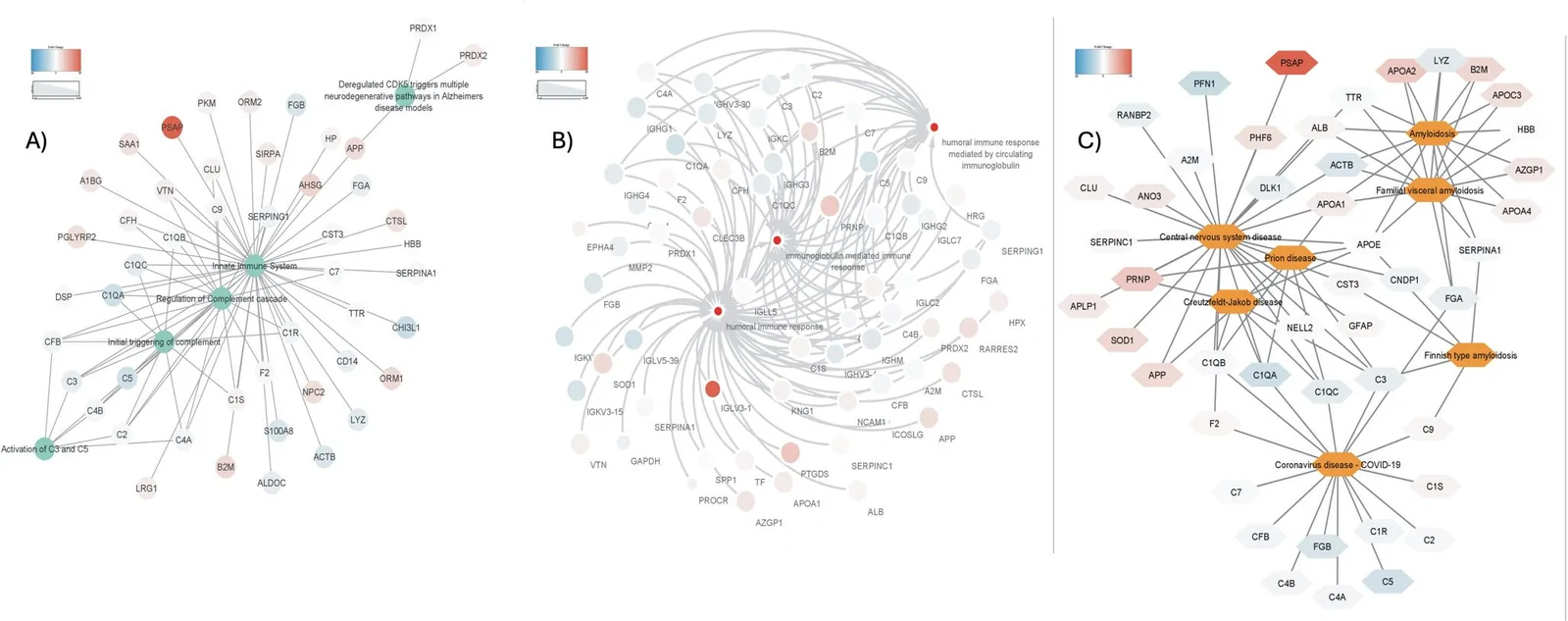
Protein–protein interaction and pathway analysis of dysregulated proteins in the CSF of a patient with multiple myeloma and SARS-CoV-2. Protein–protein interaction analysis and protein regulation in the CSF of a patient with Multiple Myeloma and SARS-CoV2 at CSF. **A** Relative levels of dysregulated proteins compared between infection and post-infectious periods enriched for innate immune system and complement cascade pathways. **B** Protein–Protein interaction analysis enriched for humoral immune response and immune globulin mediated response. Relative levels of the dysregulated proteins compared between infection and post-infectious periods and **C** Diseases enrichment analysis of proteins found dysregulated when compared between infection and post-infectious periods

Of the 184 differentially expressed proteins during infection, the majority were involved in immune system-related pathways and the regulation of the complement cascade (Figs. 4A and B). Protein–protein interaction analysis revealed significant enrichment in humoral immune response and immune globulin-mediated responses (Fig. 4B).

Notably, 16.3% (30/184) of the differentially expressed proteins were associated with neuronal function, synaptic organization, and amyloid formation. Enrichment analysis against disease databases revealed a strong association with central nervous system disorders. Additionally, protein interaction analysis indicated potential links between these proteins and those associated with COVID-19 (Fig. 4C). Among these, prosaposin, a lysosomal protein involved in sphingolipid metabolism and neuronal and glial protection, was detected exclusively during active infection [8].

**Fig. 5:**
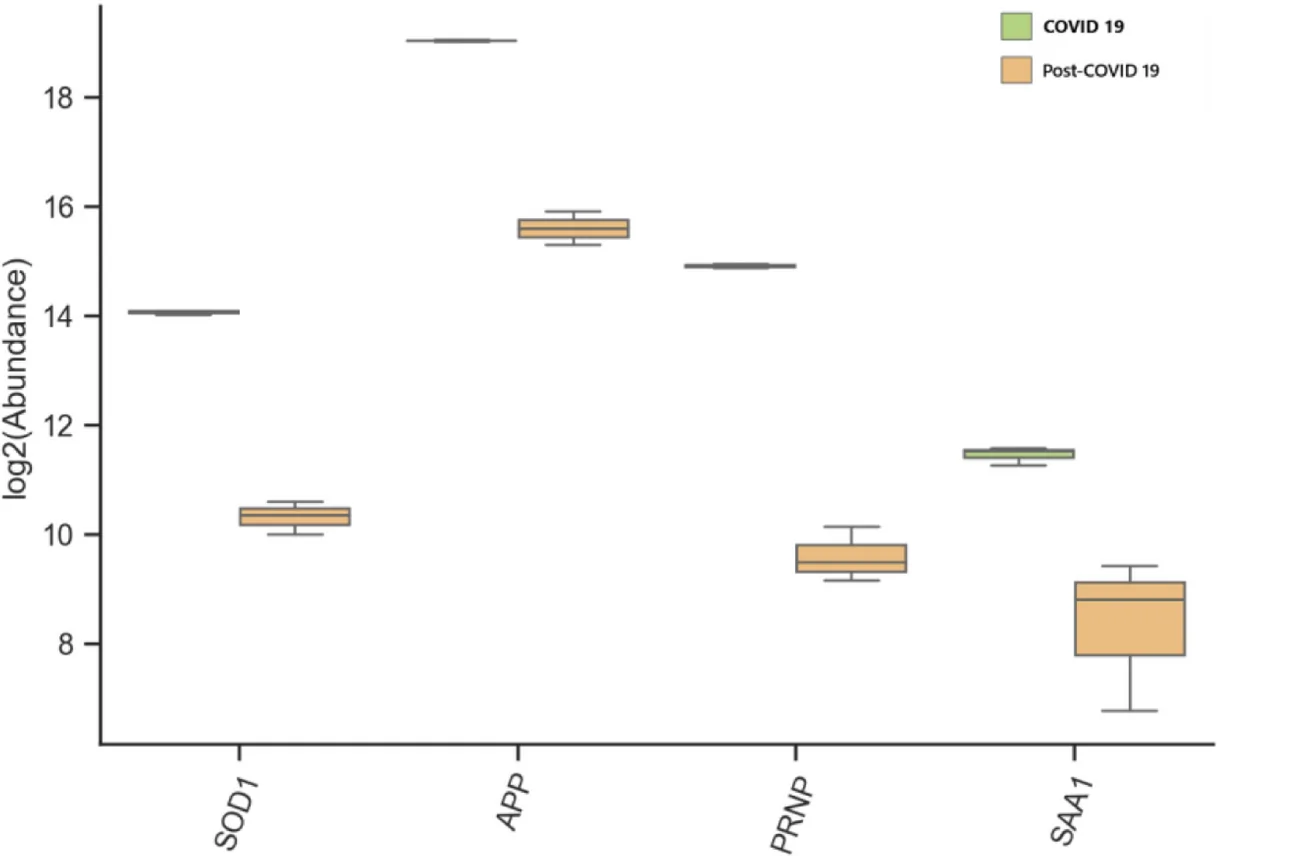
Box plot analysis of the peak intensity of peptides from SOD-1, APLP1, APP, SAA1, and PRNP/PrP in infection and post-infection periods. Box Plot analysis based on peak intensity of the sum of all peptides from Superoxide Dismutase (SOD-1), Amyloid-Like Precursor Protein (APLP1), Amyloid-ß Precursor Protein (APP), SAA1 and Prion protein (PRNP/PrP)

Several other proteins, including prion protein (PRNP/PrP), superoxide dismutase 1 (SOD-1), amyloid-β precursor protein (APP), serum amyloid A-1 (SAA1), and amyloid precursor-like protein 1 (APLP1), all related to oxidative stress, neuronal function, and amyloid formation in neurodegenerative diseases, showed substantial increases during infection compared to post-infection. Specifically, their expression levels were elevated 38, 13, 10.6, 6.8, and 4.4 times, respectively (Fig. 5).

**Fig. 6:**
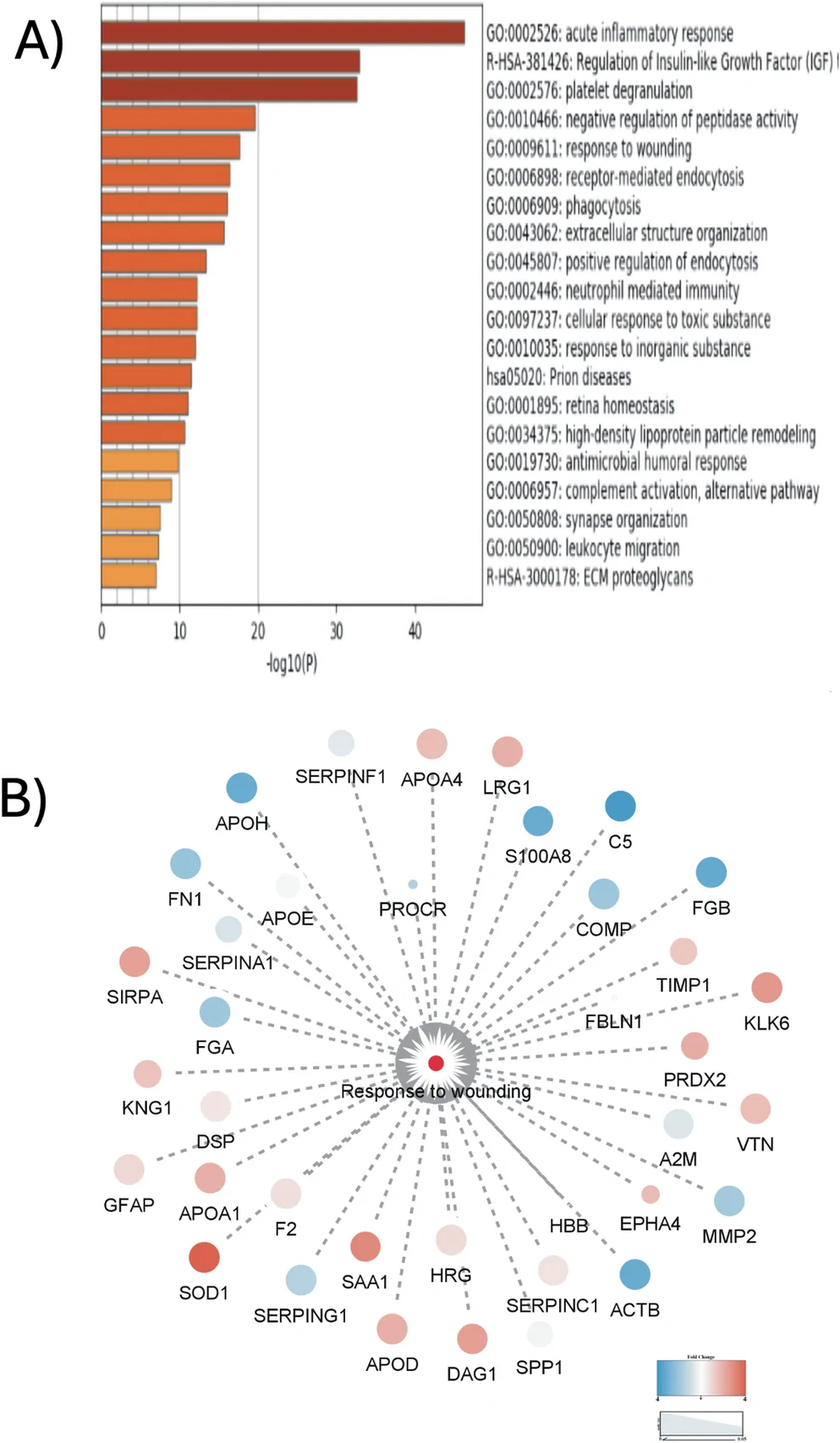
**A** Pathway enrichment analysis of dysregulated proteins between infection and post-infection periods. **B**: Protein–protein interaction and wound response pathway analysis of dysregulated proteins between infection and post-infection periods. **A** Pathway enrichment analysis of dysregulated proteins in the cerebrospinal fluid (CSF) of a patient with multiple myeloma and SARS-CoV-2 infection, comparing the acute infectious phase with the post-infectious period. **B** Protein–protein interaction (PPI) analysis and regulation of the “response to wounding” pathway, enriched among dysregulated proteins identified between the two conditions. Pathway enrichment analysis was conducted using Metascape, incorporating annotations from the KEGG, Reactome, and WikiPathways databases. The isolated “response to wounding” pathway was further analyzed using PPI networks generated with the STRING algorithm and visualized in Cytoscape. Proteins upregulated during the SARS-CoV-2 infection are highlighted in red, whereas downregulated proteins are indicated in blue

The neuroinflammatory response is further supported by analysis using the EnrichR integrative systems biology tool, which linked several deregulated proteins to wound response, indicating cell damage (Figs. 6A and B). Gene ontology analysis of the differentially expressed proteins revealed that the majority are extracellular and secreted proteins, predominantly located in vesicles. An interesting finding from the in-silico systems biology analysis, according to Reactome, was the involvement of these proteins in amyloid fiber formation, energy metabolism, and post-translational modifications (Supplementary Material).

## Discussion

Prior research on patients undergoing treatment for hematologic malignancies, including hematopoietic stem cell transplantation, has shown a heightened risk of progression to lower respiratory infections [3].

Neurological complications have been documented in various respiratory viral infections, including SARS-CoV, MERS-CoV, and more recently SARS-CoV-2 [1, 9]. Since the onset of the COVID-19 pandemic, it has become evident that SARS-CoV-2 can impact the brain either directly or indirectly [10]. Initially, neurological symptoms were primarily associated with anosmia and ageusia; however, more severe manifestations, such as encephalopathies, encephalitis, leptomeningitis, febrile or afebrile seizures, status epilepticus, and stroke, have since been reported [11]. More recently, the emergence of "long COVID" or post-COVID syndrome has raised new challenges, as symptoms like cognitive decline, memory loss, and confusion are being observed even in patients with mild COVID-19 [12, 13]. However, the specific mechanisms of neuroinvasion by the virus, or whether certain variants exhibit neurotropism, remain unclear [14]. In this case, we describe an immunocompromised MM patient who tested positive for SARS-CoV-2 in both the CSF and nasopharyngeal swabs, with no pulmonary involvement but presenting with severe neurological symptoms. Notably, the viral loads in both CSF and nasopharyngeal samples were similar, with comparable CT values between SARS-CoV-2 and RNAse-P in both sites.

MRI scans revealed hyperintense signal changes in the midbrain and pons on T2/FLAIR imaging, with less marked changes in the thalamus and corticospinal tract, consistent with rhomboencephalitis. The absence of punctiform enhancement ruled out CLIPPERS (chronic lymphocytic inflammation with pontine perivascular enhancement responsive to steroids) [15].

An additional point to consider is that the viral panel used to detect HCoV-NL63 was not performed on the CSF, as the test is not validated for this type of sample and is not replicable. There is a case report describing possible encephalitis associated with HCoV-NL63 or HKU1 in a child suggesting that neuroinvasion might be possible [16]. In our patient, HCoV-NL63 was identified only in a respiratory sample at admission, and due to technical and financial limitations, CSF testing for this virus was not feasible.

Rare conditions such as Japanese encephalitis and Dengue encephalitis can also present with brainstem involvement [17]. Acute necrotizing encephalopathy, a rare but serious complication of viral infections, has been described in association with SARS-CoV-2 and often involves symmetric, multifocal lesions in the thalamus, brainstem, and cerebellum [18]. However, our patient did not exhibit necrosis, bleeding, or significant diffusion restriction.

We performed proteomic analysis of the CSF during and after SARS-CoV-2 infection. Our findings revealed a significant inflammatory response, characterized by the presence of acute-phase proteins and regulation of immune and complement systems. The detection of serum albumin in the CSF during COVID-19 suggests blood–brain barrier disruption, which may explain the presence of acute inflammatory components [19]. These results align with previous proteomic studies showing similar inflammatory pathways in patients with neurological manifestations of COVID-19 in non-oncological settings [20]. Our data also show similarities to findings from Wang et al., with higher expression of proteins involved in the PI3K-AKT signaling pathway and cellular metabolism, consistent with the inhibition of SARS-CoV-2 entry into cells by a PI3K inhibitor [21].

We observed elevated levels of secreted pro-inflammatory cytokines in the CSF, which is consistent with findings from other studies on patients infected with SARS-CoV-2, both with and without cancer [22, 23]. The presence of inflammatory components in the CSF may indicate a neuroinflammatory process, possibly caused by the virus infection [10]. Alternatively, the disruption of the blood–brain barrier allows peripheral inflammatory mediators to enter the CSF. These two mechanisms are not mutually exclusive, and regardless of the origin, the presence of these inflammatory components in the central nervous system can trigger neuronal damage.

However, due to the inherent risks of brain parenchyma biopsy, it was not possible to obtain histological confirmation or direct evidence of viral presence in CNS tissue in this case. Therefore, while our findings suggest a potential link between SARS-CoV-2 infection and CNS inflammation in immunocompromised patients, we acknowledge that this interpretation remains hypothetical.

Interestingly, post-COVID-19 CSF showed elevated levels of osteopontin, a cytokine involved in inflammation, apoptosis, and wound healing. Osteopontin has been shown to exacerbate acute lung injury in influenza virus infection [23].

Proteins associated with amyloidogenesis and neurodegeneration, such as prion protein and amyloid A-1 protein, were highly expressed in the CSF during SARS-CoV-2 infection. The receptor-binding domain (RBD) of the S1 protein from SARS-CoV-2 binds with high affinity to amyloidogenic proteins like amyloid A1, prion protein, Aβ, tau, PD-43, and alpha-synuclein, promoting their aggregation [24].

Remarkably, we observed a 13-fold increase in the levels of superoxide dismutase (SOD-1) in the CSF during infection compared to the post-infection period. SOD-1 is known to interact with prion proteins and form macromolecular complexes with other aggregation-prone proteins, affecting microglia, astrocytes, and neurons, thus promoting cell death and neurodegeneration [25]. The role of prion proteins in inflammation is well-documented, and this mechanism may involve interactions between the nervous, endocrine, and immune systems, impacting prion protein expression and function in neutrophils [26]. The accumulation of amyloidogenic and pro-inflammatory proteins may play a significant role in the neurodegeneration observed when SARS-CoV-2 infects the nervous system.

To the best of our knowledge, the simultaneous detection of SARS-CoV-2 in both the nasal cavity and CSF has not been reported until now. Our finding of substantial viral loads in the CSF, comparable or greater than those found in the nasal cavity, has important implications. The virus's ability to infect resident brain cells, along with similar viral levels in the CNS and nasopharyngeal samples, could provide pathophysiological explanations for some COVID-19 clinical manifestations, such as anosmia and dysgeusia [13]. In addition to the direct effects of lung inflammation, CNS infection could also contribute to respiratory distress by damaging the respiratory centers in the brain.

The detection of SARS-CoV-2 at similar viral loads in both CSF and nasopharyngeal swabs, in the absence of respiratory symptoms, represents a rare and informative clinical presentation, especially considering the patient’s immunocompromised status. This observation may provide valuable insight into potential mechanisms of neuroinvasion.

Given that exacerbated inflammation can increase blood–brain barrier permeability and considering the known presence of coronaviruses in the CNS, it is possible that CSF positivity for SARS-CoV-2 may be more common than previously recognized in COVID-19 cases.This finding underscores the importance of considering neurological symptoms as part of the COVID-19 clinical spectrum when designing diagnostic and therapeutic strategies.

## Supplementary Information

Below is the link to the electronic supplementary material.

Supplementary file1 (DOCX 17 KB)

## Data Availability

The datasets used and/or analysed during the current study are available from the corresponding author on reasonable request.
