# Supplementary material for: Cerebrospinal fluid positive for SARS-CoV-2 associated with neurological manifestations and proteome alterations in a multiple myeloma patient
Source: Neurol Sci. 2026 Oct 19;47(11):871. doi: 10.1007/s10072-026-09471-w (PMC13677627; doi:10.1007/s10072-026-09471-w)
Supplement: Supplementary file 1 [file 10072_2026_9471_MOESM1_ESM.docx]

**Table 1.** Serum and Central Nervous System Laboratory Assessment at Patient Admission.

| **Initial laboratory measures** | **Reference Range, Adults*** | **On admission** |
| --- | --- | --- |
| Hematocrit (%) | 36-46 | 33.3 |
| Hemoglobin (g/dL) | 12-16 | 8.6 |
| White-cell count (per µL) | 4500-11000 | 2500 |
| Differential count (per µL) |  |  |
| Neutrophils | 1.7-7.0 x 10**^3^** | 1.5 x 10**^3^** |
| Lymphocytes | 0.9-2.9 x 10**^3^** | 0.4 x 10**^3^** |
| Monocytes | 0.3-0.9 x 10**^3^** | 0.4 x 10**^3^** |
| Platelet count (per µL) | 150,000-400,000 | 65,000 |
| Vitamin D (ng/ml) | <20 | 18 |
| Immunoglobulin levels (mg/dL) |  |  |
| IgG | 600-1500 | 424 |
| IgM | 50-300 | 17 |
| IgA | 50-400 | <5 |
| CD4 T-Lymphocytes (per mm**^3^**) | 507-1496 | 48 |
| **Diagnostic ID work up** | | |
| Respiratory multiplex PCR Panel (nasal swab) | Negative | Coronavírus NL63 |
| RT-PCR SARS-CoV-2 (nasal swab) | Negative | Positive |
| **Cerebralspinal Fluid (CSF) analysis** | | |
| White-cell count (cells/mm**^3^**) | <4 | 3 |
| Neutrophils (%) | - | 54 |
| Lymphocytes (%) | - | 30 |
| Monocytes (%) | - | 15 |
| Eosinophils (%) | - | 1 |
| Protein (mg/dL) | <40 | 20 |
| Glucose (mg/dL) | 50-80 | 76 |
| Lactate (mg/dL) | 9-16 | 20 |
| Bacterioscopy | Negative | Negative |
| Bacterial culture | Negative | Negative |
| Fungal culture | Negative | Negative |
| Adenosine Deaminase (ADA) (U/L) | <9 | <4 |
| Syphilis serology | Negative | Negative |
| Cryptococcus neoformans antigen | Negative | Negative |
| RT-PCR SARS-CoV-2 (CSF) | Negative | Positive |
| RT-PCR Herpes Virus (HSV) type 1 and 2 | Indetectable | Indetectable |
| RT-PCR Herpes Virus type 6 (HHV-6) | Indetectable | Indetectable |
| PCR Toxoplasmosis | Negative | Negative |
| RT-PCR Polyomavirus BK/ JC Virus | Indetectable | Indetectable |
| RT-PCR Adenovirus | Indetectable | Indetectable |
| RT-PCR Mycobacterium tuberculosis | Indetectable | Indetectable |
| * Reference values are affected by many variables, including the patient population and the laboratory methods used. The ranges used at AC Camargo Cancer Center are for adults who are not pregnant and do not have medical conditions that could affect the results. These reference ranges may not be appropriate for all patients. | | |
